# Effects of Taekkyon-based exercise program on balance, lower extremity strength, and gait parameters in community-dwelling older women: Randomized controlled trial

**DOI:** 10.1097/MD.0000000000037463

**Published:** 2024-03-15

**Authors:** Chang Yong Kim, Hye Won Jeong, Chang Yoon Baek, Suhng Wook Kim, Hyeong Dong Kim

**Affiliations:** a Pharma & Bio Pharma Industry Team, Department of Pharmaceutical and Bio-Pharmaceutical Industry, Korea Health Industry Development Institute, Chungbuk, Korea; b Department of Physical Therapy, College of Health Science, Korea University, Anam-ro, Seongbuk-Gu, Seoul, Republic of Korea; c Department of Rehabilitation Medicine, National Health Insurance Ilsan Hospital, Goyang-si, Gyeonggi-do, Republic of Korea; d Department of Health and Safety Convergence Science, Graduate School, Korea University, Anam-ro, Seoul, Republic of Korea.

**Keywords:** balance, elderly, gait, lower extremity strength, Taekkyon

## Abstract

**Background:**

As individuals age, they experience a decline in muscle strength and balance, leading to diminished functional capacity and an increased risk of falls. The purpose of the current study was to investigate the effects of the Taekkyon-based exercise program on balance, muscle strength, and gait ability in women aged over 65-year-old residing in the local community.

**Methods:**

Forty-eight subjects were randomly allocated into the Taekkyon-based exercise program as an experimental group (EG = 25; mean age: 71.68 ± 3.26) or a fall prevention program as a control group (CG = 23; mean age: 73.65 ± 5.88). EG participants received 1-hour Taekkyon exercise sessions twice a week for 12 consecutive weeks. CG participants received a typical fall prevention program. The measurements in each group included assessments of balance levels (the timed up-and-go test, one-leg stance, and functional reach test), lower extremity strength (the 5-chair stand test and 30-second chair stand test), and gait parameters (cadence, step length, step width, stride length, stride time, and gait velocity) before and after the intervention.

**Results:**

After the intervention, balance (timed up-and-go test, one-leg stance, and functional reach test), lower extremity strength (5-chair stand test and 30-second chair stand test), and gait parameters (cadence, stride time, and gait velocity) showed a significant improvement in EG participants compared to CG participants (*P* < .05). Compared to the normal value of balance ability and strength of elderly women over 65 years of age, most outcomes were greater than average normal values for those receiving Taekkyon exercise.

**Conclusion:**

Taekkyon-based exercise program was more effective in improving balance, lower extremity strength, and gait capacity than the usual fall prevention program in elderly women over 65 years of age. Its effects can approach normal values for women in this age group. The 12-week Taekkyon-based exercise program could be useful as part of a fall prevention program to elderly people.

## 1. Introduction

As people age, muscle strength typically weakens due to shrinkage of muscle tissue. Eccentric contraction exercise is an effective method to strengthen the muscle.^[1]^ Falls result in an estimated 10% to 25% of elderly people because of weakened sense of balance and lower extremity strength.^[2]^ The risk of falls could be lessened by increased balance that results from muscle strengthening. Increased strength of the quadriceps, the lower extremity joint knee extensor, is considered a significant factor of static and dynamic balance control in the elderly.^[3]^ Strengthening of the quadriceps muscles is an important way to lessen falls.^[3,4]^

Exercise intervention has been shown to improve balance control in the elderly and can potentially delay age-related risk factors associated with falls.^[1–4]^ Taekkyon, a Korean martial art performed using bare hands, encompasses distinctive features. One notable element is the Pumbalkki motion, a triangular walk starting from one angular point of a triangle to other joints, which is considered the fundamental and most crucial movement in Taekkyon. This involves the bending and extending of the knees, along with a rhythmic back-and-forth movement of the center of weight.^[5,6]^ The sequential lunging and squatting may induce eccentric contraction of the quadriceps muscles.^[5,6]^

Taekkyon could be effective in revitalizing the neuromuscular function of the quadriceps through repeated eccentric contractions, and as an enhancement exercise to increase dynamic balance sensory functions. Balance control, gait capacity, strength, and endurance of lower extremity are significantly improved by practicing Taekkyon.^[5,6]^ The wriggling motion with low-intensity contractions could be well-suited for elderly patients.

The potential benefits of Taekkyon exercise targeted on elderly people in terms of functional mobility and lower extremity strength has not been explored. Accordingly, the objective of the present study was to examine the impact of a 12-week Taekkyon-based exercise program on balance, lower extremity strength, and gait in elderly women residing in the local community. We hypothesized that post-intervention, the Taekkyon group would exhibit superior scores in balance, lower extremity strength, and spatiotemporal gait parameters compared to the control group (CG).

## 2. Methods

### 2.1. Subjects

Subjects were recruited from 2 public community welfare centers for elderly people in Korea, where they provide care and offer services to enhance the well-being of the community-dwelling older adults. The 60 subjects ≥65 years of age were participating in a Taekkyon-based exercise program or a fall prevention education program. Among the 60 subjects, 30 were assigned to the Taekkyon group (experimental group, EG) and 30 to the fall education group (CG). The subjects were randomly partitioned into the groups by computerized block randomization.^[7]^ Moreover, each individual participant was not given information about their group assignment. None of the participants had prior experience in Taekkyon exercise before participating in the study. The study was approved by Korea University Institutional Review Board. All of the subjects were fully informed about the objectives, methods, procedures, and risk factors of the experiment and provided written informed consent before participating.

For inclusion into the study, subjects from both groups who agreed to participate were required to fulfill the following criteria: (i) women ≥65 years of age with no history of falls in the past year, (ii) scored over 24 points out of 30 in Korean mini-mental state examination,^[8]^ (iii) scored over 45 points out of 56 in Berg balance scale, indicating not a high but still a level of fall risk,^[9,10]^ and (iv) not participating in other exercise programs or Taekkyon program for the last 6 months. The exclusion criteria included: (i) chronic disease, such as cardiovascular disease, musculoskeletal disorders, ischemic heart disease, and orthostatic hypotension and (ii) unable to walk independently without aids (cane, walker, etc) or help. Subjects knew they could stop participating at any time.

### 2.2. Equipment and data collection

The outcome measure scales for lower extremity strength, balance, and gait parameters were evaluated at pretest (baseline) and posttest (after the 12-week sessions). The same physiotherapist who conducted the outcome measurements was blinded to group assignments. Each test was repeated 2 times, with the average value recorded. Lower extremity strength measures involved the 5-chair stand test (5-CST) and 30-second chair stand test (30s-CST). The 5-CST and the 30s-CST have respectable test-retest reliability (*R* = 0.95 and *R* = 0.93, respectively) for older women.^[11]^ The validity of these tests has been demonstrated.^[12,13]^ The 5-CST is an appropriate evaluation technique to measure lower extremity strength, speed, and power. The 30s-CST is suitable to assess lower extremity strength and endurance in elderly people.^[12,13]^

The balance measures comprised the timed up-and-go test (TUG), one-leg stance (OLS), and the functional reach test (FRT). Lin et al^[14]^ examined the test-retest reliability and validity of the TUG, OLS, and FRT for older people living in the local community. The results indicated good test-retest reliability (*R* = 0.93 to *R* = 0.99), and the validity was good. Gait parameter measurements were conducted to assess spatiotemporal parameters including gait cadence, step length, step width, stride length, stride time, and gait velocity during free walking. Gait analysis data were collected by using a 6-camera motion analysis system (Vicon T10 Cameras; Oxford Metrics Ltd., Oxford, UK) at a frequency of 100 Hz, and it was low-pass filtered by a 4th-order Butterworth filter with a cutoff frequency of 6 Hz. The total of 35 markers in the Plug-in Gait Full-Body marker set were attached on subject’s various anatomical landmarks.^[15]^

### 2.3. Procedures and intervention

**Figure 1.**
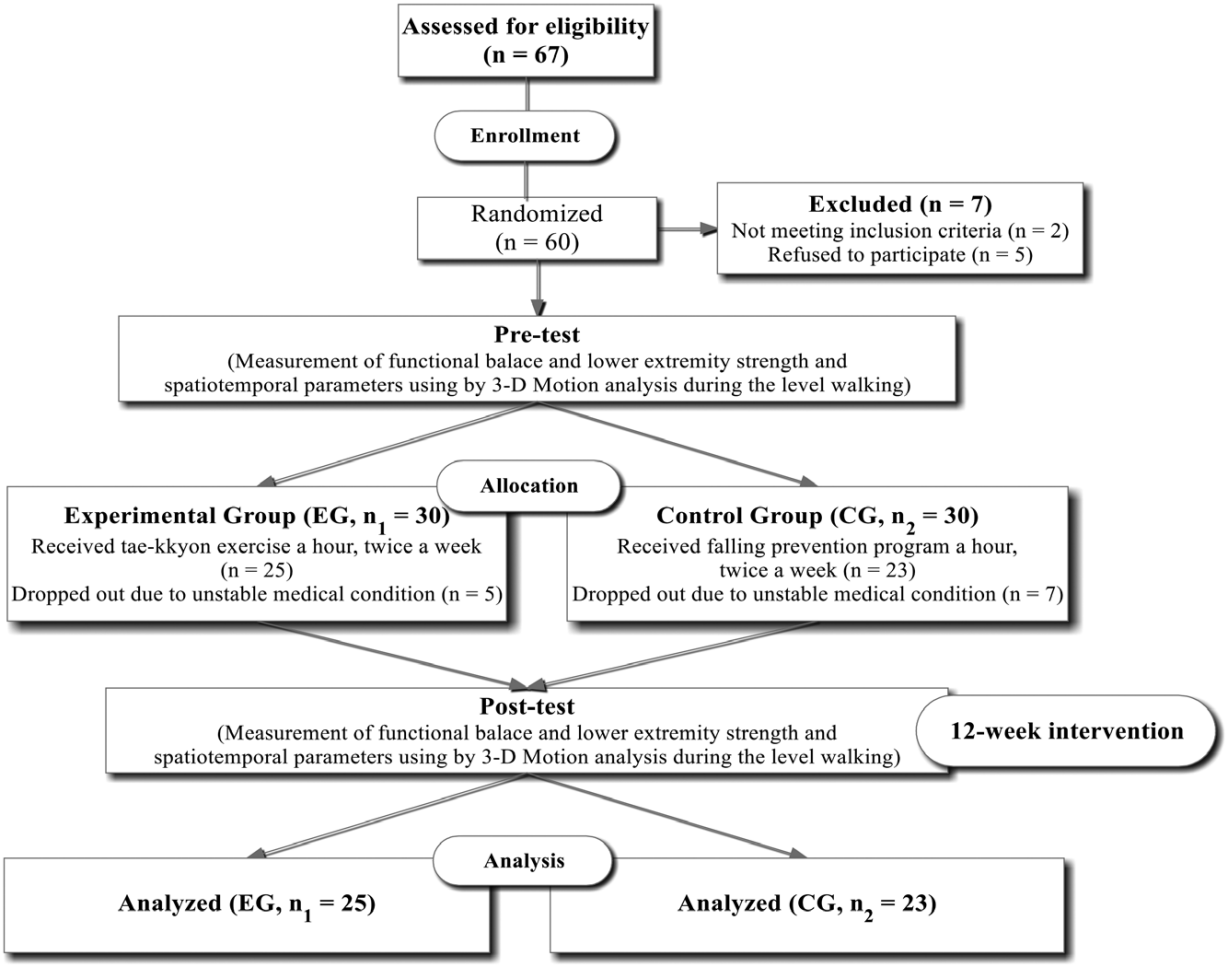
Diagram of the experimental protocol.

A flow chart of each stage is presented in Figure 1. EG participants received Taekkyon-based exercise program for 1 hour per day, 2 days per week, over 12 consecutive weeks (totaling 24 hours) at a community welfare center. All EG participants underwent training at the same intensity and duration, and a presentation occurred before the start of each exercise session, as the group training. Posttesting was conducted after each training session. CG participants received lectures delivered through a presentation and video clips on fall prevention for 1 hour per day, 2 days per week, over a period of 12 weeks (totaling 24 hours) at a local university. The Taekkyon-based exercise program was conducted under the guidance of a certified and expert-trained Taekkyon instructor who demonstrated and explained techniques established by the Korean Taekkyon Federation. Each 1-hour session followed a structured format, beginning with a 10-minute warm-up exercise. Subsequently, 40 minutes were dedicated to Taekkyon training, and the session concluded with a 10-minute cool-down exercise. The intervention programs were developed based on the findings of previous studies.^[5,6,16]^ The warm-up exercises involved body stretching to prepare participants for the main exercise session. Specifically, the warm-up incorporated kicking techniques from Taekkyon, including Ogumchagi, Mureupchagi, Yopchagi, and Apchagi (Table 1). The main Taekkyon training commenced with the Pumbalkki motion. Primarily, it involved rhythmically shifting the weight distribution between the front foot and the back foot, predominantly utilizing the knee and ankle joints. This action involved a forward and backward movement of the center of gravity, utilizing the joints of the knee and ankle. It was followed by a kicking motion that alternately raised the knee to its limit while keeping the spine erect, accompanied by spreading out the hands and rhythmically shaking the arms back and forth at 45-degree angles during the stepping. The exercises were typically performed accompanied by “Arirang,” a Korean folk song.^[5,6]^ A physical therapist with 5 years of experience joined the exercise program and served as an assistant guide throughout the entire sessions, supporting the main trainer (Table 1).

**Table 1:** Taekkyon-based exercise program.

Phase of intervention	Instruction
Warm-up exercise (10 min)	• Ogumchagi The technique involves spinning one’s body to kick the other person’s back knee joint. • Mureupchagi, The technique involves using the knee of the leg to attack the lower part of the opponent’s body. • Yopchagi It is executed by turning the body to the side and swinging the leg. • Apchagi It is lifting the foot and swiftly moving forward.
Main Taekkyon training (40 min)	• Pumbalkki It involves rhythmically shifting the weight distribution between the front foot and the back foot, predominantly utilizing the knee and ankle joints. This action involves a forward and backward movement of the center of gravity, utilizing the joints of the knee and ankle. It is followed by a kicking motion that alternately raised the knee to its limit while keeping the spine erect, accompanied by spreading out the hands and rhythmically shaking the arms back and forth at 45-degree angles during the stepping. • Hwalkaetchit During the Poombarbki technique, there is a motion of rotating the shoulder joint inward or outward. (the rhythmical shaking of upper extremities)
Cool-down exercise (10 min)	This phase concludes with overall body stretching and deep breathing.

The contents of the lecture course for CG participants were the main cause and seriousness of falls, guidance on how to take emergency steps, and how to relieve emotional aspects associated with falling. The video clips focused on the prevention of falls and the effectiveness of preventative measures, the necessity, and effectiveness of regular exercise. The lectures included details on materials related to falls that could occur in any setting, ways to enhance surroundings to minimize the chance of falls, a diet to reduce the likelihood of dizziness, and effective exercises for fall prevention, such as Otago, Tai Chi, and Taekkyon.

### 2.4. Data processing and statistical analyses

Statistical analysis of the data was performed by using SPSS software version 12.0 (SPSS Inc., Chicago, IL). Analyses were performed on an intention-to-treat principle; thus, all available data from all subjects were included in the analysis. The paired *t* test was used to compare the changes between pretest and posttest measurements within each group. For the comparison between groups, an analysis of covariance (ANCOVA) was conducted, with covariates set as pre-intervention variables. Statistical significance was set at a *P* value < 0.05. A sample size of 17 subjects in each group was calculated by statistical power analysis using the following values: error = 0.05, power = 0.8, effect size = 0.7, on the basis of OLS test conducted in the previous study on the effect of Tai chi exercise program,^[15]^ G*power version 3.1.3 (Franz Faul, University Kiel, Germany).^[17]^

## 3. Results

A total of 60 subjects were initially enrolled in the study. Over the 12-week experimental period, 5 subjects from the EG and 7 subjects from the CG dropped out, due to changes in their private existing medical histories, resulting in dropout rates of 16.7% and 23.3%, respectively (Fig. 1). The data of those who dropped out were excluded from the analyses. Out of the these, 48 completed all interventions and assessments, with 25 in the EG and 23 in the CG. Throughout the study, no adverse events, such as falls, were reported. Additionally, there were no significant differences in demographic characteristics between the groups (Table 2).

**Table 2:** Subject characteristics for each group.

Characteristics	Experimental group (EG = 25)	Control group (CG = 23)	*t*	*P* value
Age (yr)	71.68 ± 3.26	73.65 ± 5.88	−1.42	.17
Height (cm)	150.14 ± 4.84	150.41 ± 7.30	−0.15	.88
Weight (kg)	57.34 ± 8.20	58.86 ± 10.56	−0.56	.58
K-MMSE (score)	29.04 ± 1.06	28.83 ± 1.15	0.67	.51
BBS (score)	55.0 ± 1.15	54.52 ± 1.31	1.34	.19
BMI (kg/m^2^)	25.44 ± 3.35	25.85 ± 3.32	−0.42	.68

Values are expressed as the mean ± standard deviation.

BBS = Berg balance scale, BMI = body mass index, K-MMSE = Korean mini-mental state examination.

### 3.1. Balance and lower extremity strength

The overall changes in the balance and lower extremity strength are listed in Table 3. At the baseline test, there were no significant differences between the groups from TUG, FRT, OLS, 5-CST, and the 30s-CST. After the experimental session, the EG participants exhibited significant improvements in balance and lower extremity strength measures, from the TUG, FRT, OLS, 5-CST, and the 30s-CST (*P* < .05), but not in CG.

**Table 3:** Comparisons of clinical changes of the balance and lower extremity strength measures between the 2 groups.

Characteristics	Experimental group (EG, n_1_ = 25)		Control group (CG, n_2_ = 23)		*F*	*P* value†
	Pretest	Posttest	Pretest	Posttest		
Balance						
Timed up and go (s)	10.12 ± 1.73	9.08 ± 1.24*	10.71 ± 1.74	11.10 ± 1.82	28.00	<.001
Functional reach (cm)	27.53 ± 2.73	31.82 ± 4.46*	27.76 ± 4.63	28.83 ± 4.32	6.01	.02
One-leg stance (seconds)	19.46 ± 16.42	26.98 ± 17.66*	13.52 ± 17.15	13.15 ± 16.78	11.67	<.01
Lower extremity strength						
5-chair stand test (s)	10.57 ± 2.73	9.02 ± 2.58*	11.14 ± 2.61	10.58 ± 2.16	5.95	.02
30s-chair stand test (number)	17.06 ± 5.95	21.04 ± 7.96*	15.67 ± 4.09	16.08 ± 4.58	9.30	<.01

Values are expressed as the mean ± standard deviation.

* Within-group comparison by paired *t* test, *P* < .05.

† Between groups comparison by analysis of covariance with pre-intervention variables as covariates.

**Figure 2.**
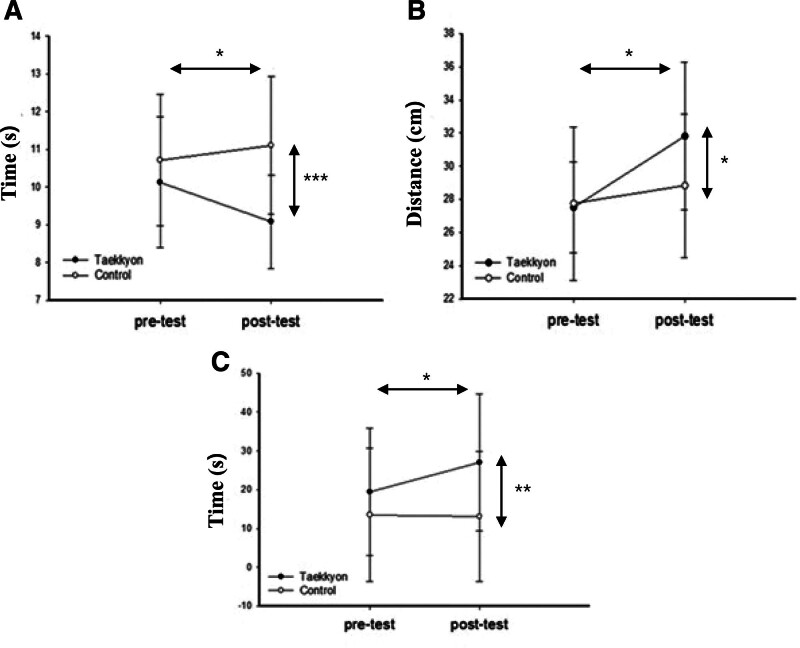
Comparison of Taekkyon and control groups on balance measures of (A) timed up-and-go test, (B) functional reach test, and (C) one-leg stance test before and after the intervention. Values are mean ± standard deviation. *P < .05, **P < .01, and ***P < .001.

**Figure 3.**
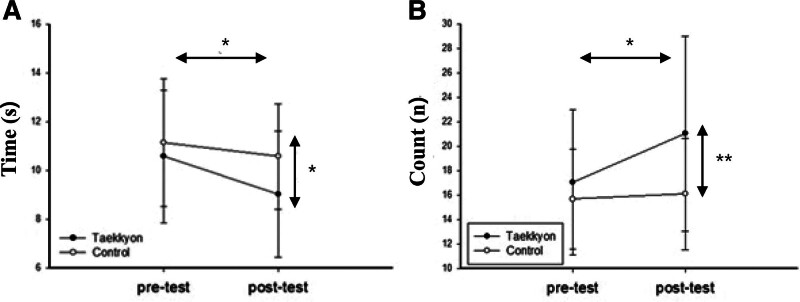
Comparison of Taekkyon and control groups on lower extremity strength measures of (A) 5-chair stand test and (B) 30s-chair stand test before and after intervention. Values are mean ± standard deviation. *P < .05 and **P < .01.

In the comparison between groups using ANCOVA, the EG participants demonstrated significantly greater improvement in the values from TUG (*F*_1,47_ = 28.0, *P* < .001), FRT (*F*_1,47_ = 6.009, *P* < .05), OLS (*F*_1,47_ = 11.670, *P* < .01), 5-CST (*F*_1,47_ = 5.949, *P* < .05), and 30s-CST (*F*_1,47_ = 9.303, *P* < .01) compared to the CG (Figs. 2 and 3).

### 3.2. Spatiotemporal gait parameters during the level walking

**Figure 4.**
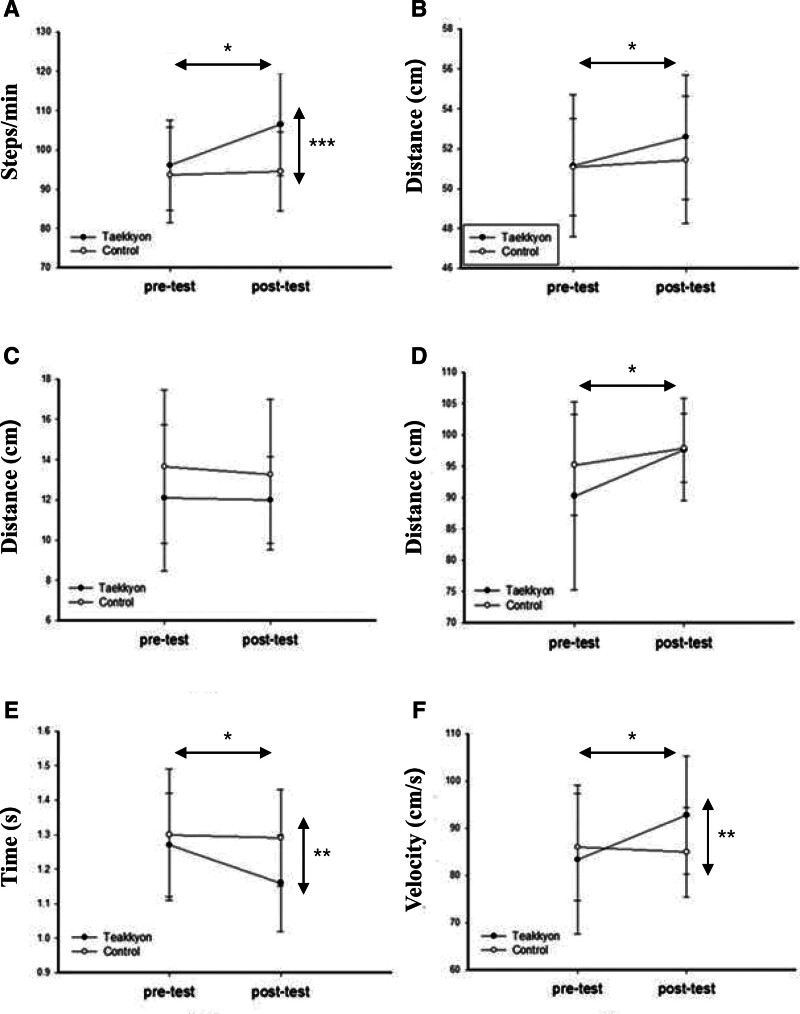
Comparison of Taekkyon and control groups on spatiotemporal parameters of (A) cadence, (B) step length, (C) step width, (D) stride length, (E) stride time, and (F) gait velocity before and after the intervention. Values are mean ± standard deviation. *P < .05, **P < .01, and ***P < .001.

Table 4 presents the overall changes in spatiotemporal gait parameters during the level walking in each group. At the pre-intervention stage, significant differences were observed between the 2 groups, with the EG participants exhibiting a higher cadence, while the CG participants demonstrated a longer stride length and faster gait velocity. After the intervention, the EG participants exhibited significant improvements in cadence, step length, stride length, stride time, and gait velocity (*P* < .05), but not in CG. In the comparison between groups with ANCOVA, there were significant changes, with higher improvement in cadence (*F*_1,47_ = 13.262, *P* < .001), stride time (*F*_1,47_ = 11.744, *P* < .01), and gait velocity (*F*_1,47_ = 11.718, *P* < .01) in the EG participants compared to the CG participants (Fig. 4).

**Table 4:** Comparison of the gait performance for the 2 intervention groups.

Characteristics	Experimental group (EG = 25)		Control group (CG = 23)		*F*	*P* value†
	Pretest	Posttest	Pretest	Posttest		
Cadence (steps/min)	96.12 ± 11.48	106.5 ± 13.10*	93.58 ± 12.15	94.53 ± 10.03	13.23	<.001
Step length (cm)	51.14 ± 3.57	52.59 ± 3.12*	51.08 ± 2.43	51.44 ± 3.20	1.95	.17
Step width (cm)	12.10 ± 3.65	12.00 ± 2.15	13.66 ± 3.82	13.26 ± 3.73	0.48	.49
Stride length (cm)	90.27 ± 15.02	97.67 ± 8.2*	95.20 ± 8.06	97.9 ± 5.47	0.22	.64
Stride time (s)	1.27 ± 0.15	1.16 ± 0.14*	1.30 ± 0.19	1.29 ± 0.14	11.74	<.01
Gait velocity (cm/s)	83.34 ± 15.69	92.78 ± 12.46*	86.01 ± 11.30	84.92 ± 9.49	11.72	<.01

Values are expressed as the mean ± standard deviation.

* Within-group comparison by paired *t* test, *P* < .05.

† Between groups comparison by analysis of covariance with pre-intervention variables as covariates.

## 4. Discussion

The study was designed to clarify the effect of Taekkyon-based exercise program on balance, lower extremity strength, and gait capacity of women ≥65 years of age living in the local community and to provide basic data for developing Taekkyon for inclusion in a fall prevention program incorporating exercise. To our knowledge, this is the first randomized controlled trial comparing a supervised Taekkyon-based exercise program with a typical fall prevention education program concerning fall prevention within a rehabilitation program specifically designed for older women in the local community.

### 4.1. Balance measures

After the Taekkyon exercise sessions, the EG participants demonstrated significant improvement in the TUG, FRT, and OLS tests compared to the CG participants. This aligns with previous finding that indicated a significant improvement in the TUG test (pretest: 8.12 s; posttest: 6.45 s) for elderly individuals aged ≥70 years after a 4-week Taekkyon exercise program.^[18]^ A study by Lim reported a significant improvement in the FRT test (pretest: 30.9 cm; posttest: 33.2 cm) among subjects aged 60–80 years who participated in structured Taekkyon Pumbalkki movements 3 times a week for 12 weeks.^[16]^ Another study demonstrated a 4-week Taekkyon exercise program led to improvements in TUG, FRT, and OLS for older women aged ≥70 years.^[6]^

Taekkyon motion incorporates postures that require a 1-legged stance during dynamic movements of the body’s center of gravity in various directions (e.g., Pumbalkki) to execute a diverse range of kicking techniques, achieved through subtle knee flexion or extension.^[6,16]^ Essentially, Taekkyon is designed to enhance the body’s dynamic balance by necessitating controlled shifts in the center of gravity along the anterior-posterior and lateral planes.^[6]^ Taekkyon exercise exerted an impact on momentum generation by augmenting the magnitude of center of pressure movement in the anterior-posterior and medial-lateral directions, contributing to the overall maintenance of balance.^[5,6,16]^ Furthermore, change values on TUG and OLS tests of EG group following intervention achieved reference and normal values with 9.2 seconds in TUG and the 20.3 to 60 seconds in OLS for corresponding age group.^[19,20]^

### 4.2. Lower extremity strength measures

Those who received Taekkyon exercise showed significant improvements in the 5-CST and 30s-CST, consistent with the findings of previous studies.^[6,18]^ The Pumbalkki motion in Taekkyon exercise is characterized by executing rhythmic triangular step movements, alternating knee flexion and extension, and dynamically shifting body weight.^[16]^ This movement, similar to a semi-lunge and semi-squat, is sufficient to activate the muscles in the knee joint area.^[6]^ A previous study has reported that Taekkyon exerts a significant impact on vertical ground reaction force, leading to the activation of quadriceps muscles.^[16,18]^ Furthermore, the changes in values observed in the 5-CST and 30s-CST of the EG group following the intervention surpassed both the reference and normal values, registering at 12.6 seconds in 5-CST and 17 repetitions in the 30s-CST for the corresponding age group. These change values correspond to those of individuals aged 60 to 69 years.^[19,21]^

### 4.3. Spatiotemporal parameters during the level walking

After the Taekkyon exercise sessions, the EG participants showed a general improvement in gait capacity. Furthermore, compared to the CG, EG intervention led to significant improvements in gait parameters such as cadence, stride time, and gait velocity. This result is consistent with previous findings,^[6]^ indicating that both Taekkyon exercise and Tai Chi exercise led to improvements in gait capacity in community-dwelling older women. The results also showed that Taekkyon training had a similar effect to Tai Chi training on gait capacity. The effectiveness of Tai Chi training has been elucidated in many studies to improve walking in older adults and people with neurological disorders.^[6,22–24]^ In other words, the results of our study will help demonstrate Taekkyon-based exercise can improve walking ability in older adults. The Taekkyon-based exercise has the advantage of combining lower extremity strength training in the ankle and knee joints with dynamic balance training.^[6,16,18]^ It encompasses all the necessary elements, such as balance training and strengthening approaches, to sufficiently improve the walking function of elderly people.^[25–27]^ Additionally, as demonstrated by the results in balance (TUG, FRT, and OLS) and lower extremity strength (5-CST and 30s-CST), the EG improved both balance and lower extremity strength through Taekkyon-based exercise. Therefore, we infer that the concurrent improvement in lower extremity strength and balance may have contributed to enhancements in the gait of older adults.^[6]^

Gait velocity stands out as a paramount variable among gait parameters. A higher gait velocity signifies a more pronounced enhancement in functional mobility among elderly female subjects. This significance is underscored by a study reporting a clinically meaningful change in gait velocity of approximately 5 cm/s, with a substantial, meaningful change noted at 10 cm/s in the elderly subjects.^[6,28]^ Namely, the Taekkyon-based exercise program can produce clinically significant changes in gait velocity in elderly women.

### 4.4. Study limitations

This study has several limitations. First, generalizing the results may be challenging due to the small sample size, despite having calculated for it. Furthermore, as the study was conducted exclusively in women over the age of 65, caution should be exercised in extending the findings to men. Second, the absence of a follow-up test prevents us from determining whether there was a retention effect. Furthermore, we did not include outcome measures related to falling, such as the fall efficacy scale, the number of falls, or corresponding follow-up observations. Third, the lack of research on the subject contributes to a dearth of comparable studies. Fourth, the CG did not receive comparable interventions, such as strengthening and balance training similar to those in the EG, suggesting a potential lack of equity in the intervention. In future studies aiming to better understand the efficiency of Taekkyon-based exercise program, researchers should take these aspects into consideration.

## 5. Conclusions

The Taekkyon-based exercise program was more effective in improving balance, lower extremity strength, and gait capacity than the usual fall prevention program in elderly women over 65 years of age. Its effects brought these parameters closer to normal values for women in this age group. The findings suggest that the 12-week Taekkyon-based exercise program could be useful as part of a fall prevention program to elderly people.

## Acknowledgments

Authors are grateful to all subjects involved in this study as well as authors/publishers/editors of all those articles, journals and books from where the literature for this article has been reviewed and discussed.

## Author contributions

**Conceptualization:** Chang Yong Kim, Hye Won Jeong.

**Data curation:** Suhng Wook Kim.

**Formal analysis:** Suhng Wook Kim.

**Investigation:** Chang Yong Kim, Hye Won Jeong.

**Supervision:** Chang Yoon Baek, Hyeong Dong Kim.

**Validation:** Hyeong Dong Kim.

**Writing – original draft:** Chang Yong Kim, Hye Won Jeong, Chang Yoon Baek.

**Writing – review & editing:** Hyeong Dong Kim.
